# Detection and analysis of photo-acoustic emission in Direct Laser Interference Patterning

**DOI:** 10.1038/s41598-021-93927-w

**Published:** 2021-07-15

**Authors:** Tobias Steege, Sabri Alamri, Andrés Fabián Lasagni, Tim Kunze

**Affiliations:** 1 Fraunhofer-Institut für Werkstoff-und Strahltechnik IWS, Winterbergstr. 28, 01277 Dresden, Germany; 2 Fusion Bionic GmbH, Löbtauer Str. 69, 01159 Dresden, Germany; 3 Institut für Fertigungstechnik, Technische Universität Dresden, George-Bähr-Str. 3c, 01069 Dresden, Germany

**Keywords:** Surface patterning, Acoustics, Characterization and analytical techniques, Lasers, LEDs and light sources, Photoacoustics

## Abstract

Functional laser texturing by means of Direct Laser Interference Patterning is one of the most efficient approaches to fabricate well-defined micro textures which mimic natural surfaces, such as the lotus effect for self-cleaning properties or shark skin for reduced friction. While numerous technical and theoretical improvements have been demonstrated, strategies for process monitoring are yet to be implemented in DLIP, for instance aiming to treat complex and non-plane surfaces. Over the last 35 years, it has been shown that the sound pressure generated by a laser beam hitting a surface and producing ablation can be detected and analysed using simple and commercially available transducers and microphones. This work describes the detection and analysis of photo-acoustic signals acquired from airborne acoustic emission during DLIP as a direct result of the laser–material interaction. The study includes the characterization of the acoustic emission during the fabrication of line-like micro textures with different spatial periods and depths, the interpretation the spectral signatures deriving from single spot and interference ablation, as well as a detailed investigation of the vertical extent of the interference effect based on the ablated area and its variation with the interference period. The results show the possibility to develop an autofocusing system using only the signals from the acoustic emission for 3D processing, as well as the possibility to predict deviations in the DLIP processing parameters.

## Introduction

Laser based tools for micromachining and surface functionalization provide today enormous potentials for the processing of various materials. In particular, the selective change in the surface topography is playing an increasingly important role to enhance the surface properties of many products or devices, for example reducing the adhesion of ice^1,2^, modifying the friction^3^ or tuning the wettability of a surface^4^. Moreover, laser micro-processing is replacing conventional methods in many other areas such as welding of materials^5^, drilling of boreholes^6^ and cutting of thin material^7^. As part of this replacement process, approaches for process monitoring and control have been established. For instance, in the field of laser welding and drilling, process monitoring includes the analysis of the mid- and long wave infrared radiation via infrared cameras or pyrometers for investigating the heat development, CCD and CMOS cameras to capture process lighting, as well as microphones for recording the process noise^8^.

These monitoring approaches are nowadays slowly adapted into functional laser texturing, especially for already well-established approaches such as Direct Laser Writing (DLW), where a single laser beam is scanned over the material surface, creating microstructures through the ablation process^9^. An advanced technology for the texturing surfaces with micro- and nanoscale structures is Direct Laser Interference Patterning (DLIP) and consists in coherently superimposing two or more laser beams to create a periodic interference pattern within the laser spot size. The interference pattern can be defined by the wavelength and polarization of the laser light, the number of beams and their intersection angles, and allows a large degree of freedom in surface design^10,11^. Furthermore, this texturing approach has already been demonstrated on a variety of material such as copper^12^, ceramics^13^ and glass^14^ and can be consider suitable for any material which can be treated by laser.

In both mentioned approaches, the working position and the size of the laser spot are critical process parameters, which have a significant influence on the final result and even shifts of a few micrometres in the working position can lead to deviations in the process results^15^. In particular, for the 3D processing of components and parts, an optimal process control of the working distance is fundamental^16,17^. Besides the direct approach of splitting the laser beam and measuring the partial beam with a sensor^18,19^, optical detectors can also monitor the process from a lateral position. The low light generation in micro laser machining with pulsed lasers and the positioning of the photodetector, at small working distance complicates this approach^8^. In contrast to optical methods, acoustic methods offer both the advantage of a non-contact measurement and the possibility to set the system relatively far away from the working position. As a result, several studies for evaluating the airborne or surface acoustic emission (AE) during the laser micromachining process have been conducted^20,21^. Evgueni et al.^22^ were able to show that the surface-acoustic emissions in copper foils can be recorded with a contact microphone, retrieving relevant process information such as the change in pulse energy. However, the results showed that process monitoring based only on the AE is still challenging due to the complex non-linear ablation processes and the underlying time-varying statistical and AE spectral signatures. Furthermore, the quality of the structure-borne emission was found to depend on the distance between the origin of the emission source and the contact microphone, and can only be recorded on the surface of the material. In a later work, a correlation between change of the focus position to the work piece and AE was shown^23^. Similarly, the airborne AE can be utilized as demonstrated by Weber et al.^24^, for finding the focus position for direct laser processing with picosecond laser pulses, whereby an additional visual evaluation with a CCD camera was used for achieving higher accuracy. Advanced solutions based on real-time multi-sensor systems are already available for quality monitoring, e.g. to measure the surface roughness^25^.

Different approaches for process monitoring in DLIP have been also investigated. It has been demonstrated that the heat accumulation after texturing can be monitored by measuring the heat radiation with a high speed infrared camera in an off-axis position. It was shown that for materials like stainless steel and aluminium the quality of the structure can be correlated with the thermal effects^26^. However, if the heat accumulation on the surface is not substantial, either due to the material properties or the heat input of the process itself, no signals can be detected through this method. On the other hand, an optical system for quality inspection has been developed, which takes advantage of the back reflection of the light diffraction of periodic textures. Through this method, different patterns (e.g. line- and dot-like pattern) can be analyzed and the retrieved signals can be correlated with the quality of the microstructures^27^. However, due to the nature of its working principle, microstructures which tend not to reflect light (e.g. due to surface oxidation or large structure depth) can be hardly detected through this optical method. Complementary to this technique, scatterometry can also be utilized as quality monitoring for surfaces functionalised through DLIP^28^. Also in this case, the quality of the measurement depends on the amount of reflected light. It has to be mentioned that all the above-mentioned and explored process monitoring approaches for DLIP, are based mainly on the evaluation of the quality of a periodic pattern and do not gain any information from the ablation process itself.

The objective for this work, is to demonstrate the utilization of the AE method as a possible candidate for process monitoring in DLIP. In particular, the informational properties carried by the signal are examined and a correlation of the obtained information with the shape of the interference volume is performed.

## Results and discussion

### Analysis of the AE by single-pulse ablation

**Figure 1:**
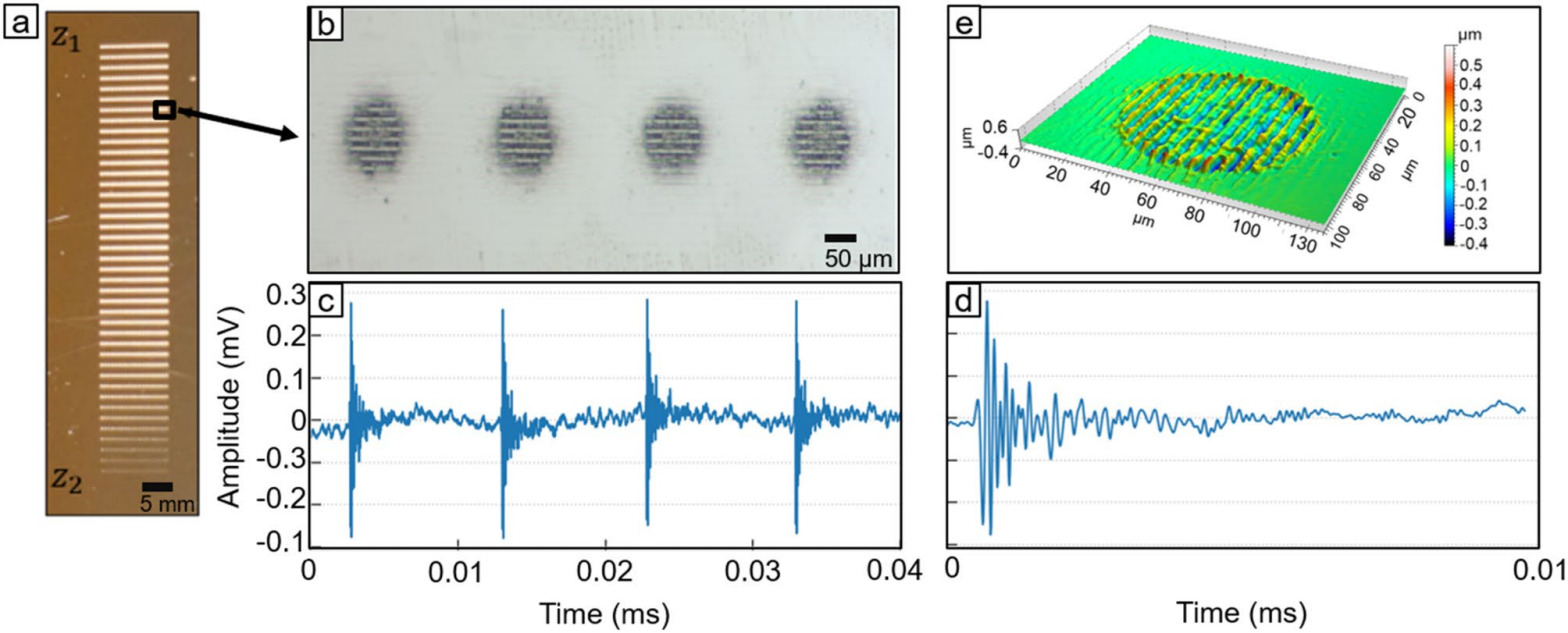
(**a**) Laser lines at different z1 working position to z2 to the focus length of the lens (**b**) Laser spot with interference pattern (**c**) Sound profile of four laser spots (**d**) Sound profile of one laser spot (**e**) Ablated spot with interference.

The DLIP setup employed in this work is a compact optical module consisting of diffractive, refractive and focusing optics, by which a main infrared laser beam can be split into two sub-beams and microscopic line-like patterns with variable size (spatial period) can be generated. The last element of the module is a focusing lens, which both focusses and overlaps the sub beams on the material’s surface. The system is designed in order to let the sub-beams to intersect approximately 3.6 mm above the focal plane of the focusing lens. This configuration has the advantage to increase the available size of the interference volume (both in height and section) as well as reducing the variation of the spatial period depending on the later position. Furthermore, the setup avoids to concentrate the total available pulse energy in a confined area, making the process more stable and at the same time allowing a better control of the surface topography. The description of the setup can be found in the Materials and Methods section and the working principle has been explained elsewhere^11^. In order to acquire acoustic signals during the texturing processes, a microphone has been placed at 50 mm from the ablation position and several lines consisting of separated pulses have been marked on a flat stainless steel plate (Fig. 1a). Each line consists of 10 laser pulses (Fig. 1b) marked at the same working position and, while moving to the successive line, the line was marked at a different working position, with a total variation of 1 mm from the known position of interference volume (Z = 37.2 mm), i.e. the position where the two interfering beams cross each other producing an interference pattern. As it can be seen in Fig. 1b, each pixel contains a line-like pattern, characteristic of a two beam DLIP process (Fig. 1e).

As commonly known for nanosecond laser surface texturing, depending on the laser fluence, the interaction of each laser pulse with the sample surface can result in melting, vaporization of material, ablation, and formation of a vapour-plasma plume, and thus shock waves in the material’s surface as well as and in the surrounding air are formed^29–31^. Thus, the shock waves propagate through the air and reach the microphone, where the resulting airborne AE is transduced into electric signals that can be acquired and analysed. Figure 1c shows an example of the temporal evolution of the acoustic signal while marking one line at a repetition rate of 1 kHz, using a pulse energy of 255 µJ. The time between each peak is 1 ms, which corresponds to the employed laser repetition rate. It can be noted that the baseline of the acoustic signal slightly varies in the amplitude, which can be ascribed to environmental noise, in particular to the cooling fans of the laser and the mechanical motion of the linear axes. Furthermore, Fig. 1d shows the audio signal of a single laser pulse. Of particular interesting is the length of ablation signal, which is less the 1 ns and, in contrast to the pulse length of 15 ns, showing that the shockwave of the laser-interaction is recorded and is only a fraction of the total laser pulse. This indicates that the removal of material and the generation of a plasma plume take place in the very first instants of the irradiation event. The mentioned 1 ns duration of the sound wave, is generated when the absorption length of the material is overcome and a laser supported detonation (LSD) occurs. The underlining effect and a detailed analyses of the velocity of the propagation of the shockwave and its properties can be found elsewhere^32,33^.

To obtain the characteristic information of the AE from the ablation of one laser pulse, a spectral analysis in the frequency domain was performed by applying a Fast Fourier Transformation over the signal^34^. The spectral analysis for various spatial periods can be found in the supplementary section (Fig. S2). From this, the maximum sound pressure (MSP) is calculated for the frequency range of 18 kHz to 22 kHz applying a Butterworth bandpass filter^35^, and converted to sound pressure (in Pa) taking into account the sensitivity of the microphone. An analysis of the frequency band for single laser ablation can be found here^32^. The MSP is expressed dB of the effective pressure in relation to the sound pressure in air. Previous research have shown that this range is unaffected by the ambient and machine noise and has the amplitude intensity for analysis which is not given by the lower frequencies^36^.

**Figure 2:**
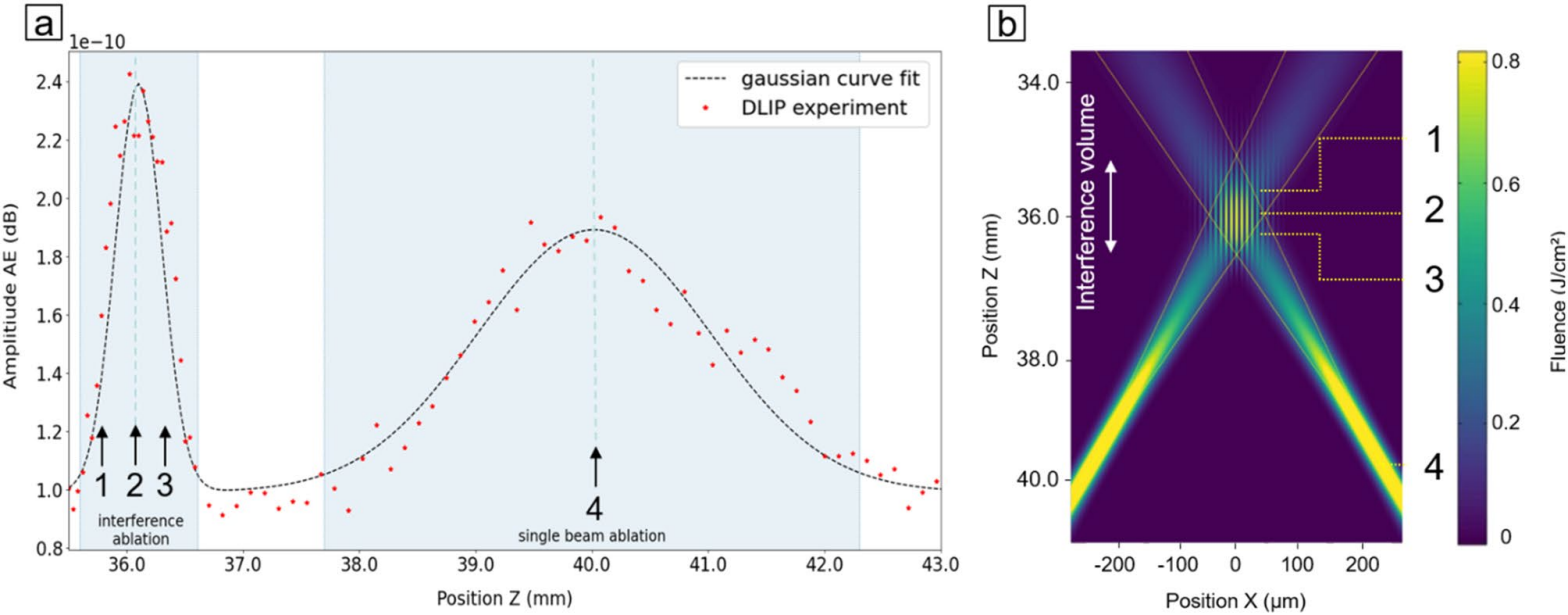
(**a**) Change of the AE signal with respect of the surface z position in relation to the laser head, (**b**) Simulation of the intensity of the interference volume generated by two focused laser beams.

In a second set of experiments, the ablation process reported in Fig. 1 has been repeated varying the working position 2.5 mm above the known interference position and 3.5 mm below the focal length of the aspheric lens, with the aim to analyse the AE for an interference setup also beyond the position of the interference volume (i.e. the working position). Specifically, a pulse energy of 255 µJ and a spatial period of 8.0 µm were set, while the working position has been varied with a step of 0.04 mm, collecting 3500 audio files from 350 different Z-positions and 10 laser pulses per position. As a result of the experiment (Fig. 2a), the AE signals achieve a relative maximum value in two positions, namely 36.40 mm and 40.00 mm, which can be attributed to the optimal working position for the interference patterning process and the focus position of the single sub-beams after the interference volume, respectively.

In order to obtain the characteristics of these signals, the collected data points have been fitted with two Gaussian functions (Fig. 2a) which, in case of the interference volume, also matches the assumptions of the shape generated by superimposing Gaussian beams^37^. As a result, the FWHM of the interference volume peak (0.65 mm) can be considered as an estimation of the extent of the interference volume. On the other hand, the AE peak related to the focus position of the sub-beams shows a larger (FWHM of 3 mm) and a less intense emission. This can be explained taking into consideration that this AE peak is a result of the incoherent addition of two sound sources with same intensity. In fact, it is known that he incoherent sum of two distinct sound sources (I1 and I2) depends on the logarithm of the product of both intensities instead of the direct sum of I1 and I2^38^. This difference demonstrates the complexity of emission generation during laser ablation, as one could assume that identical laser pulses penetrating the material at the same time would result in identical AEs and thus direct addition of the sound sources. On the other hand, the difference in FHWM of the single beam ablation can be attributed to the separation of the beams, as the energy density of the individual laser pulse outside of the focal length is lower than the material damage threshold. Thus, the emission peak of the DLIP region is limited within some hundreds of micrometres, while for the separated sub-beams it ranges within almost four millimetres. This means that, looking at the integral of the AE signal, the separated sub-beams have a higher AE energy (integral intensity).

In order to graphically explain the AE results, the beams overlap region of the employed DLIP setup has been simulated and represented in the YZ-plane, as shown in Fig. 2b. Further information regarding the simulation can be found in the Supplementary Materials section of this work. In this simulation, four areas are of interest can be identified, namely (1) a beams overlap area above the position where the beams fully intersect, (2) the position where the beams intersect (called the working position of the interference volume), (3) the overlap area below the working position and (4) the focus position of the individual beams. In particular, the laser beams cross each other with an angle *θ* = 7.10*°*, leading to a spatial period of 8.0 µm, and the largest interference area (position 2) is reached at 36.40 mm. Furthermore, these beams are focussed 3.6 mm underneath the interference volume.

### Characteristics of the interference-generated AE

**Figure 3:**
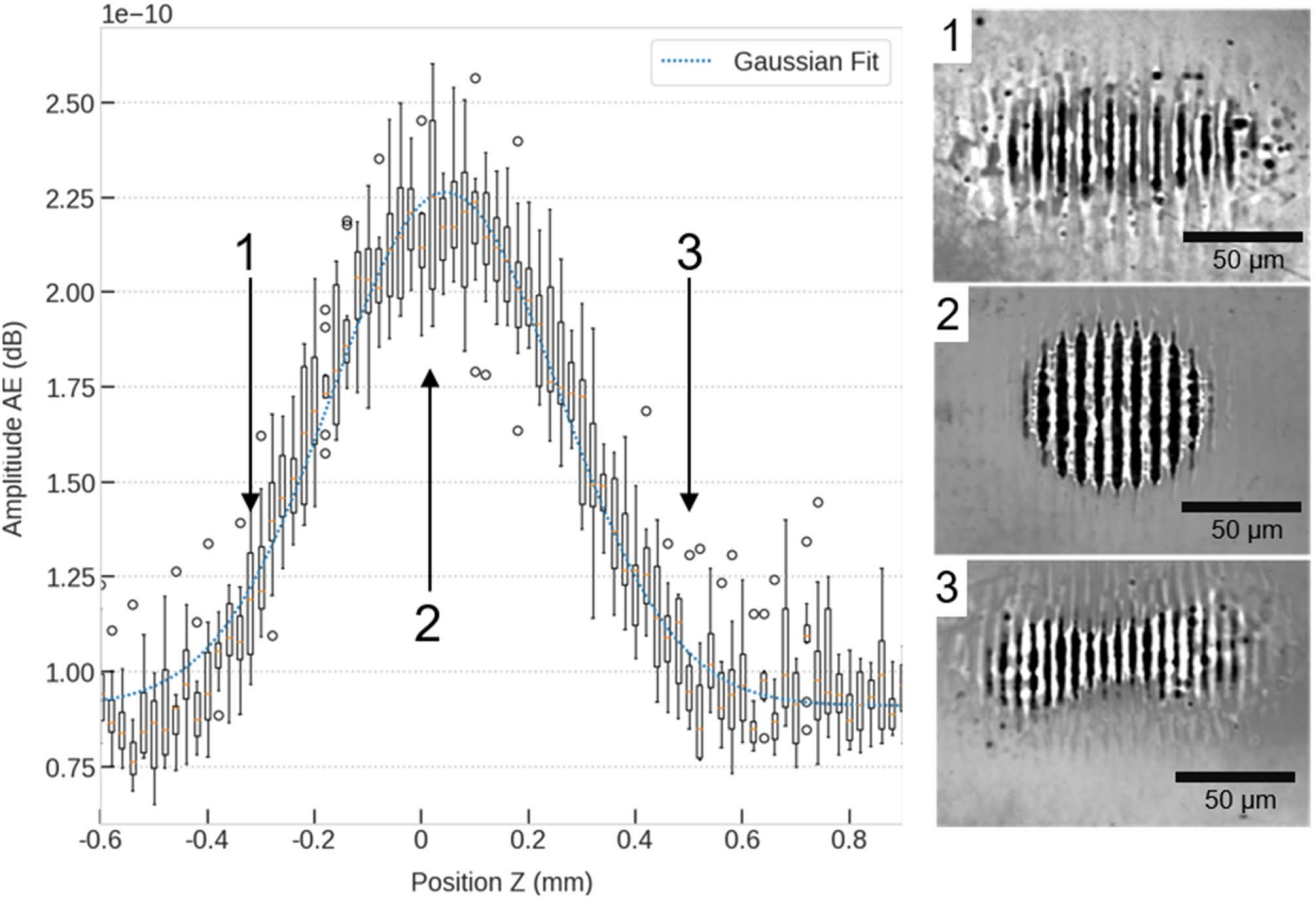
Absolute maximum sound pressure level during DLIP processing for varying z distance. The optimum working position of the interference volume is 36.15 mm.

Once the approximate position of the interference volume has been found by analysing the acoustic emission of the DLIP system (Fig. 2), further experiments have been carried out to determine the key-characteristics of the AE within the interference volume and their variation with the change of the interference period. Taking as a reference the position corresponding to the maximum of the interference-generated AE (36.4 mm), further ablation experiments with smaller step width were performed varying the working position 1 mm above and below this reference value, applying a spatial period of 8.0 µm and a pulse energy of 255 µJ. In particular, varying the working position with a step of 0.02 mm, 76 audio files have been collected and analysed in batch, resulting in the curve reported in Fig. 3. The maximum of the curve at 36.4 mm can be confirmed as the actual central position of the interference volume, shown as zero on the X-axis of the diagram (Fig. 3—Position 2). In order to provide statistical significance to the measurement, the experiment was repeated ten times and the skewness of the measurement (boxplot in the diagram) together with the outliers (dots in the diagram) have been considered for the evaluation of the retrieved data shown in Fig. 3. Also, in this case, the median of the collected data has been fitted with a Gaussian fit in order to extract the relevant parameters for investigation, such as FWHM, intensity and position of the maximum. As a result, it can be noted that the Gaussian fit correctly describes the evolution of the AE in the interference volume (R^2^ = 0.97) and that minor deviations can be observed in the outer regions of the interference volume (i.e. below -0.4 mm and beyond 0.4 mm) due to the asymmetry of the interference volume caused by the slight focussing of the interfering beams^39^. This can also be seen in the shape of the ablated laser spot on the material. As above the working position at (1) no distinction can be made between the individual beams, in contrast to below where the shape of the individual beams can be distinguished see (3). At the working position (2), the ablation spot is identical to the Gaussian distribution of the laser beam used.

**Figure 4:**
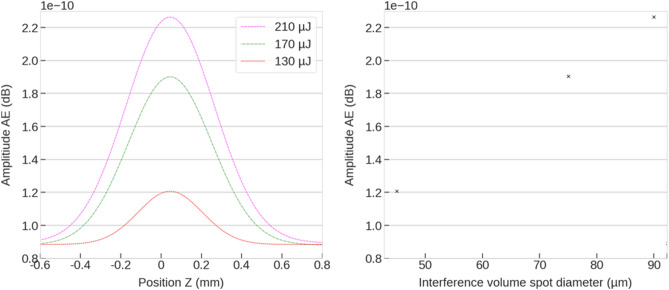
(**a**) Absolute maximum sound pressure level during DLIP processing for varying laser energy and z positions for spatial period of 8 µm. (**b**) Resulting interference spot diameter for pulse energies of 130, 170 and 210 µJ.

Figure 4a shows the evolution of the AE for different pulse energies as function of the z-position. As it can be observed, for increased pulses energies a higher amplitude in the AE was measured. This is consistent with literature observations, where it was observed that the resulting shock wave, and thus the AE amplitude, is related to the size of the ablated laser spot^20^. This relationship can be observed in Fig. 4b, where a linear correlation between the spot diameter at the material surface and the amplitude of the acoustic emission (in dB) was measured. Please note, that each dot in the figure corresponds to the maximal amplitude for the use pulses energies of 130, 170 and 210 µJ.

**Figure 5:**
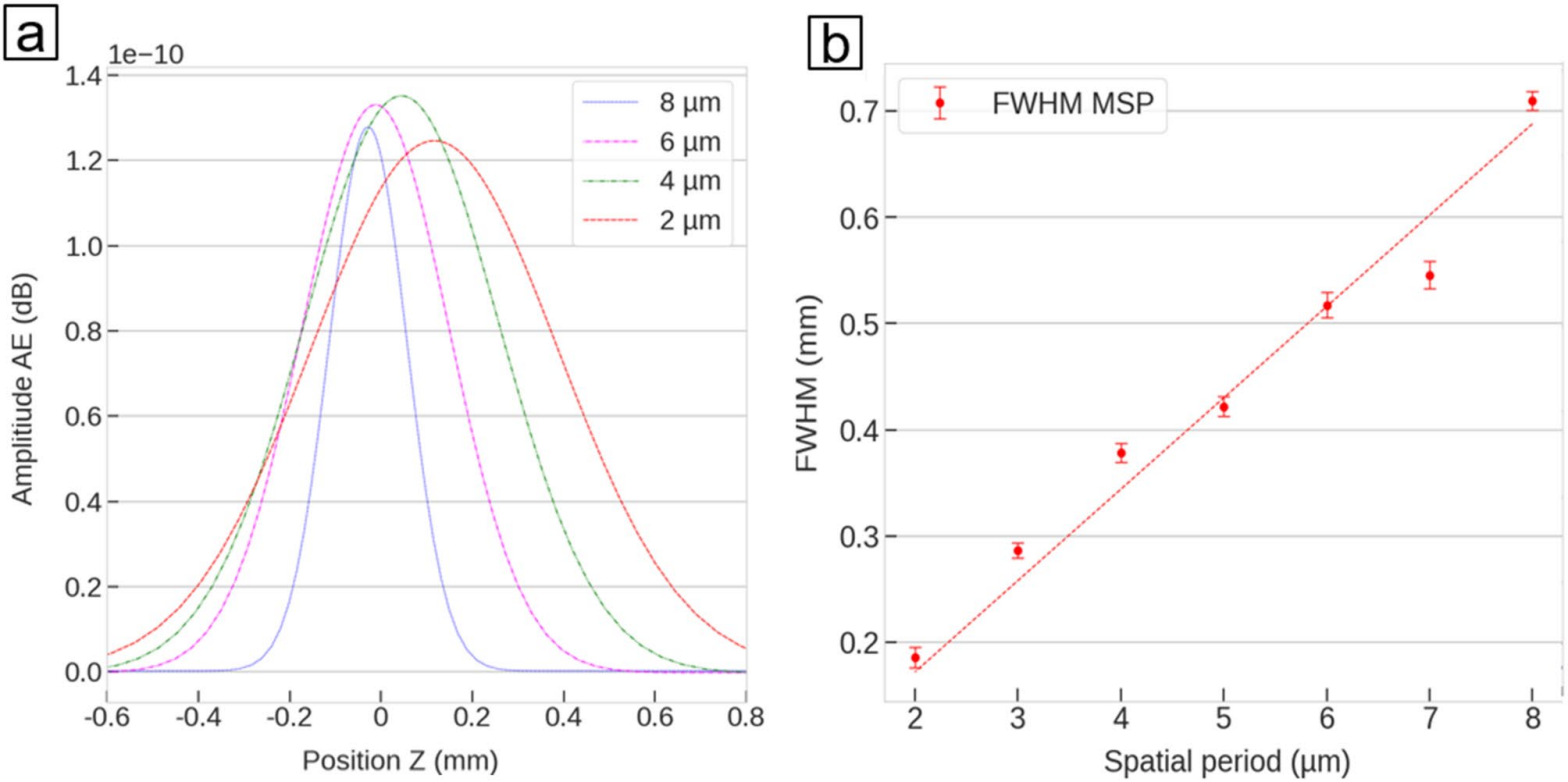
(**a**) MSP for different spatial periods, 8 µm, 6 µm, 4 µm and 2 µm approximated by a Gaussian function, (**b**) Change of the FWHM of the approximated sound curve for pulse energy 210 µJ.

In successive experiments, the AE deriving from the ablation using different spatial periods has been evaluated repeating the same analysis described in Fig. 3 by a fluence of 210 µJ. These results are presented in Fig. 5, where Fig. 5a shows the acoustice sweep for different spatial periods approximated by Gaussian function with the same pulse energy, and Fig. 5b shows the calculated FWHM as a function of the applied spatial period.

The analysis of the results allows the following remarks:

1. The full width half maximum (FWHM) of the AE can be assumed to be an estimation of the size (height) of the interference volume and it systematically decreases for decreasing spatial periods.
2. The amplitude of the AE varies applying different spatial periods.
3. The working position shifts upwards (smaller Z-values) with decreasing spatial periods, i.e. with increasing angles between the interfering beams.

The observation (1) can be explained taking into account that the height of the intersection volume between the two Gaussian beams decreases with increasing the interference angle (i.e. with decreasing spatial periods). As it can be noticed, the relationship between size of the interference volume and spatial period can be assumed to be linear (Fig. 5b). This observation is in agreement with the theoretical description developed by Brayton^37^. In particular, the height of the interference volume $\Delta z$ can be correlated to the laser beam waist (*w*) at the interference volume and the angle $\alpha$ between the crossing beams as follows:

1 $$\Delta z=\frac{4w}{sin\alpha}$$

Substituting the interference angle $\alpha$ with its definition as a function of the spatial period, Eq. (1) can be expressed as a linear relation between height of the interference volume and spatial period (*Λ*), as follows:

2 $$\Delta z=\frac{2w}{\lambda}\Lambda $$

where *λ* is the laser wavelength.

Applying this equation in order to fit the datapoints in Fig. 5b, a laser beam diameter (2*w*) of 87 µm can be retrieved, which matches the known beam waist size measured with a beam camera (~ 90 µm).

**Figure 6:**
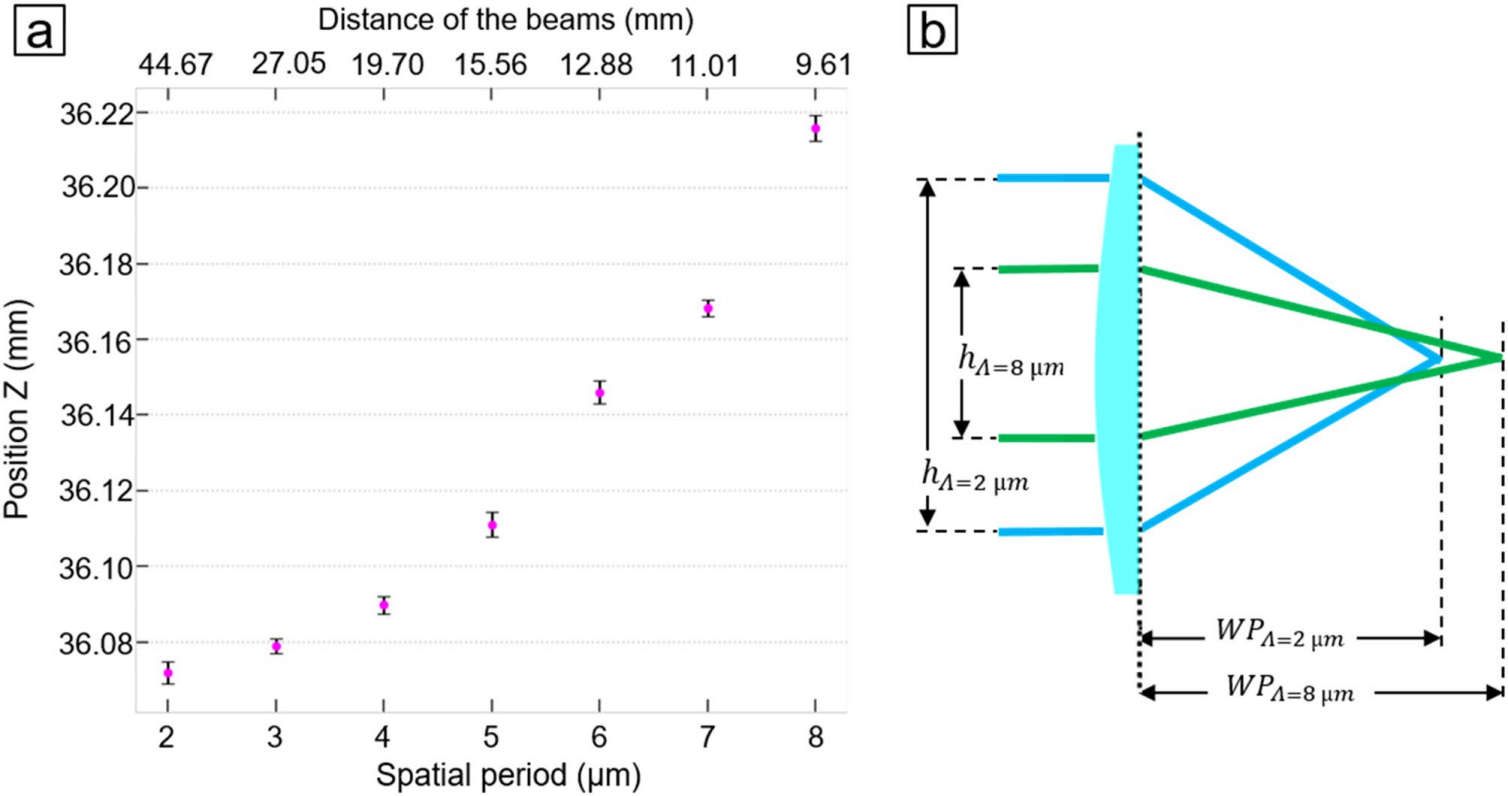
(**a**) Shift of the working position of the interference volume by changing the spatial period for pulse energy 255 µJ, (**b**) Illustration of the working position shift for two different spatial periods.

The variation of the AE intensity mentioned in the observation (2) can be attributed to two possible effects, which must be considered when the spatial period is changed. Firstly, when two laser beams are overlapped under a certain angle, an elliptical laser spot is produced, which can be approximated to a round spot for small intersection angles (e.g. 1–10°). However, when the angle is increased in order to produce interference patterns with smaller spatial period, the spots clearly show an elliptical shape. Thus, when the spatial period is changed from 8 to 2 µm (corresponding to intercepting angles of 7.1 and 31.7°, respectively), the size of the laser beam following the direction perpendicular to the interference lines increases by approximately 10 µm for the configuration used (~ 11.1%). Therefore, larger amounts of material can be ablated due to the increased spot’s area, resulting in higher AE levels. On the other hand, it is known for DLIP using ns pulses, that for smaller spatial period, the observed temperature at the maxima positions decreases do to heat diffusion^40^. In consequence, the amount of ablated material can decrease. In consequence, we have to competitive effects which could partially explain the observed differences. Additional experiments are necessary in the future to understand this effect. As far as observation (3) is concerned, the vertical shift of the interference volume can be described through simple optics assumptions. A systematic analysis of this shift as a function of the spatial period is shown in Fig. 6a and resembles a parabolic dependence. This shift can be attributed to the aspheric lens used for focusing the beams and the effect of the residual longitudinal spherical aberration by varying the lateral distance between the interfering beams^41^. This effect is shown in Fig. 6b for a low spatial period (green) and for a large period (blue). In addition, the shift also depends on the angular deviation of the beams, this can be neglected as an aspheric lens used in the setup.

## Conclusions

This work presents a systematic investigation of Acoustic Emission (AE) generated during single-pulse ablation experiments in Direct Laser Interference Patterning (DLIP). As a first objective, the response of the acoustic transducer to the ablation events has been investigated and a correlation between the retrieved data and the optical characteristic of the interference setup used has been found. In particular, it was found that the working position of the interference volume and the ablation of the single beams can be distinguished from each other. The clear change of the sound level obtained through the variation of the Z-positions can be therefore employed to identify the optical interference working position. Moreover, the analyses performed on the AE curves permitted to retrieve characteristic information about the size and shape of the interference volume, as well as the shift of its position as a function of the spatial period. An indication of the accuracy of this approach can be seen in the change in AE in contrast to the amount of interference volume extracted from the acoustic sweep. For a spatial period of 2 µm, the step size was ten times smaller than the FWHM. The next study will focus on the AE induced by the change of other process parameters, such as pulse overlap and hatching distance, with the aim to correlate the acoustic signals with information about structures depth or structure quality. A detailed understanding is necessary to enable the development of an AE autofocus to automatically maintain the working distance at a defined position when processing non-planar surfaces. Furthermore, the transfer of this approach to different materials, such as copper and ceramics, or to materials which can be treated without inducing ablation, will be investigated in future experiments.

## Materials and methods

### Material selection

Flat samples made of X5CrNi18-10 stainless austenitic chromium nickel steel (EN 1.4301/AISI 304) with a thickness of 0.7 mm and dimension of 55 mm × 85 mm were used for the experiments. The surfaces have a surface roughness Sa of ~ 70 nm (electro-polished; measured according DIN-ISO 25178) and all surfaces were cleaned from contaminations prior to the laser treatment using ethanol. The material was chosen due its relevance in several industrial sectors (e.g. food industry^44^ or aerospace^42,43^).

### Experimental setup and direct laser interference patterning

**Figure 7:**
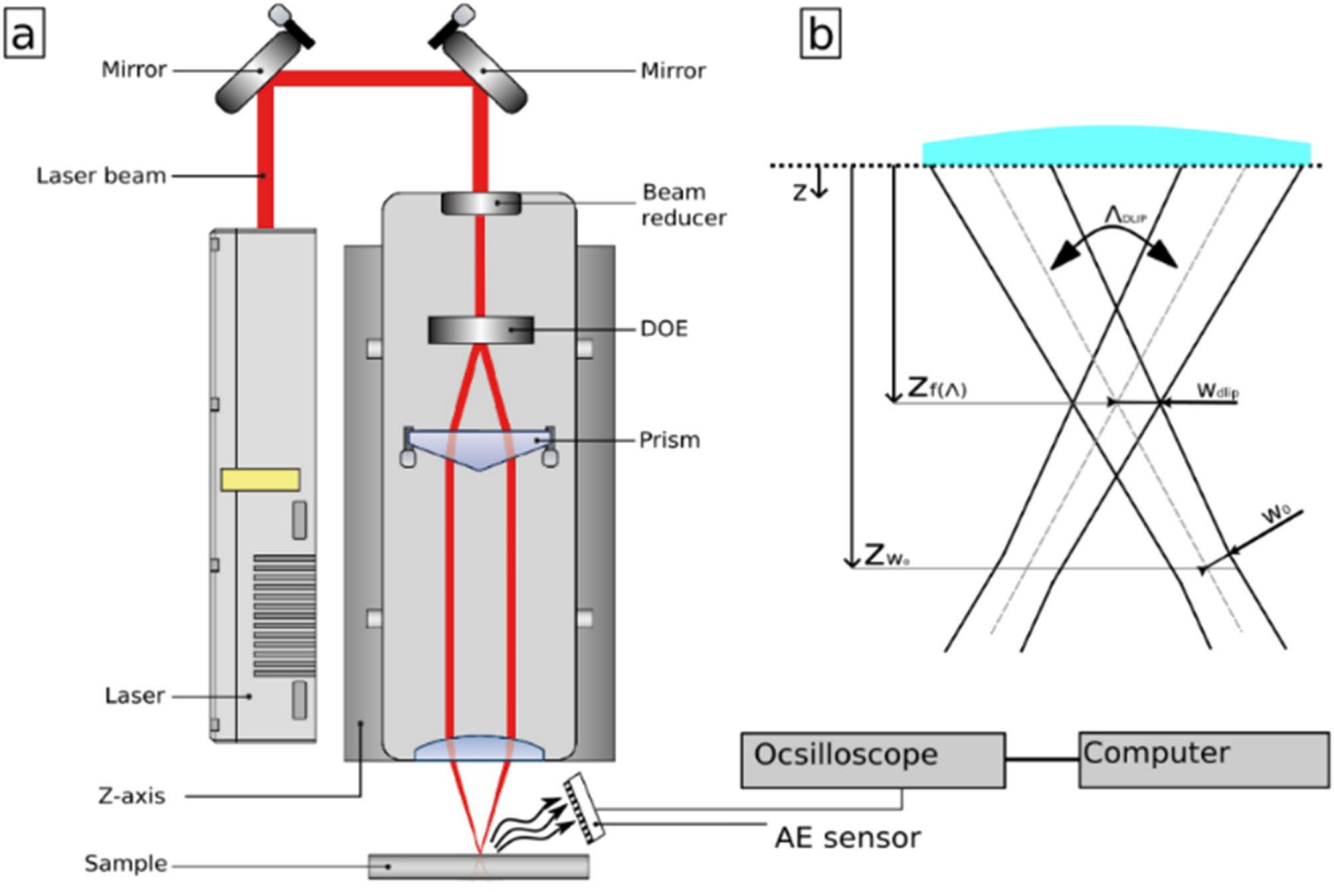
(**a**) DLIP setup with a laser system with the attached electret microphone (the microphone was placed on the back of the DLIP module so that both beams are symmetrical to the sensor), (**b**) Geometry of a DLIP Volume.

Figure 7a shows the experimental setup for both, Direct Laser Interference Patterning (DLIP) and the acoustic process measurement. In the present setup, a coherent laser beam is split into two partial beams, which are superimposed on the work piece in a controlled manner. In this way, an interference pattern can be obtained which can be used for material processing creating periodic structures. The periodicity of the interference pattern can be controlled by the overlapping angle change, which directly depends on the distance between prism and lens. More details on the relationship between spatial period and overlapping angle can be found elsewhere^45^. For the experiments, the DLIP-µFab system (Fraunhofer IWS, Dresden, Germany) in a two-beam configuration was used, where the change of the spatial period, workpiece movement and laser control is done by the surface structuring software SurfaceMod (Fraunhofer IWS, Dresden, Germany). The used laser source is a Q-switched Nd:YLF laser (Laser export Tech-1053 Basic, Moscow, Russia) with a wavelength of 1053 nm generating 15 ns pulses with a pulse energy of up to 290 µJ (@1 kHz).

For the AE measurements, an omnidirectional electret condenser microphone CMA-454PF-W (CUI Devices, Lake Oswego, USA) connected to a MAX4466 (maxim, San Jose, USA) preamplifier was installed next to the DLIP processing head and aligned on the back of the DLIP module so that both beams are symmetrical to the sensor. The AE audio sensor has a frequency bandwidth of 20 Hz to 22 kHz, a sensitivity of − 44 dB, a diameter of 10 mm and a height of 5 mm and was mounted diagonally above the laser interaction zone at a distance of 50 mm (see Fig. 7a). The preamplifier was set to a gain of 25 × (200 Vpp). The components were selected according to their specification for later integration into a compact monitoring module. The audio data was acquired with a digital oscilloscope (Analog Discovery 2, Pullman, USA, maximum sampling rate of 100 MS/s). The continuous audio signal was triggered with a frequency of 400 kHz and a sampling rate of 2.5 µs before the laser pulse and both amplitude and time response of the signal were recorded. During the AE measurement, a time series of 200 µs was recorded for each lase pulse, which corresponds to 8.000 data points to represent a laser pulse.

The AE signal was recorded for every laser pulse impinging on the material, while moving high precision motorized axes (Aerotech, PRO165LM, Pittsburgh, USA) at a speed of 5 mm/s and producing separated ablation areas containing a microscopic interference pattern (DLIP-pixels). The measurement of the DLIP working position and the focus of the single beams was performed by varying the Z-position of the DLIP processing head (see Fig. 7b). The interference angles were varied between 31.70° and 7.10° for the fabrication of line-like patterns with spatial periods between 2.0 µm and 8.0 µm. The spatial period can be calculated as follows^11^

3 $$\Lambda=\frac{2\lambda }{sin\alpha }$$

where $\lambda$ is the wavelength of the laser and $\alpha$ is the included angle the laser beams.

## Supplementary Information

Supplementary Information.
