# Supplementary material for: Detection and analysis of photo-acoustic emission in Direct Laser Interference Patterning
Source: Sci Rep. 2021 Jul 15;11:14540. doi: 10.1038/s41598-021-93927-w (PMC8282666; doi:10.1038/s41598-021-93927-w)
Supplement: Supplementary file 1 — Supplementary Information. [file 41598_2021_93927_MOESM1_ESM.docx]

Detection and analysis of photo-acoustic emission in Direct Laser Interference Patterning

Tobias Steege^1,*^, Sabri Alamri^1^, Andrés Fabián Lasagni^1,2^ and Tim Kunze^1^

^1^ Fraunhofer-Institut für Werkstoff- und Strahltechnik IWS, Winterbergstr. 28, 01277 Dresden, Germany.

^2^ Institut für Fertigungstechnik, Technische Universität Dresden, George-Bähr-Str. 3c, 01069 Dresden, Germany.

^*^ tobias.steege@iws.fraunhofer.de

**Supplementary Material**

*Simulation of the interference of two laser beams*

In the following, a more extended treatment for the propagation of two focussed interfering Gaussian beams is proposed, taking as a reference the wok of Brayton^1^ and including the focusing of the single interfering beams.

The intensity of a propagating wave is defined as the time-averaged magnitude of the Poynting flux $\left\langle\left. S \right\rangle\right.$, expressed as:

|  | $\left\langle\left. S \right\rangle\right.=\frac{1}{2} Re(E\times H^{*})$ | 3 |
| --- | --- | --- |

where, due to the superposition principle, $E=E_{1}+E_{2}$ and $H^{*}=H_{1}^{*}+H_{2}^{*}$. Equation 3 can be rewritten as:

|  | $\left\langle\left. S \right\rangle\right.=\frac{1}{2Z_{0}}Re(E_{1}\cdot E_{1}^{*}e_{1}+E_{2}\cdot E_{2}^{*}e_{2}+E_{2}\cdot E_{1}^{*}e_{1}+$  $-E_{2}\cdot e_{1}\cdot E_{1}^{*}+E_{1}\cdot E_{2}^{*}e_{2}-E_{1}\cdot e_{2}\cdot E_{2}^{*}).$ | 4 |
| --- | --- | --- |

In the explicit form, considering two waves with same polarisation ($\beta_{1}=\beta_{2}$) and equal amplitude ($E_{1}=E_{2}$), Equation 4 can be written as:

|  | $\left\langle\left. S \right\rangle\right.=e_{z}\frac{1}{2Z_{0}}\cos\frac{\theta}{2}\left[ E_{1}E_{1}^{*}+E_{2}E_{2}^{*}+2Re\left( E_{1}+E_{2}^{*} \right) \right]$ | 5 |
| --- | --- | --- |

where $Z_{0}=\frac{1}{\varepsilon_{0}c_{0}}$ is the impedance of free space. Each wave can be defined using the ($x,y_{j},z_{j}$) Cartesian coordinate system as follows:

|  | $E_{j}=E_{j0}\exp\left[ -\frac{1}{{w_{o}}^{2}}\left( x^{2}+y_{j}^{2} \right) \right]\times\exp\left[ i\omega t-i\frac{2\pi z_{j}}{\lambda}+i\varepsilon_{j} \right]$ | 6 |
| --- | --- | --- |

with $\omega$ and $\varepsilon_{j}$ are optical frequency and initial phase, respectively, and $w_{o}$ is the Gaussian beam waist, that is the radial distance at which the beam’s intensity falls to $1/e$ of its peak value. Expressing the amplitude distribution of the two beams can be expressed in terms of the power of each and transforming the coordinates system in $(x,y,z)$, the following equation can be retrieved for the electric fields:

|  | $E_{1}=\left( \frac{4Z_{0}P_{1}}{\pi{w_{o}}^{2}} \right)^{\frac{1}{2}}\exp\left\{ -\frac{1}{{w_{o}}^{2}}\left[ x^{2}+\left( y\cos\frac{\theta}{2}-z\sin\frac{\theta}{2} \right)^{2} \right] \right\}\times\exp\left[ i\omega t-i\frac{2\pi}{\lambda}\left( z\cos\frac{\theta}{2}+y\sin\frac{\theta}{2} \right)+i\varepsilon_{1} \right]$  $E_{2}=\left( \frac{4Z_{0}P_{2}}{\pi{w_{o}}^{2}} \right)^{1/2}\exp\left\{ -\frac{1}{{w_{o}}^{2}}\left[ x^{2}+\left( y\cos\frac{\theta}{2}-z\sin\frac{\theta}{2} \right)^{2} \right] \right\}\times\exp[i\omega t-i\frac{2\pi}{\lambda}(z\cos\frac{\theta}{2}+y\sin\frac{\theta}{2})+i\varepsilon_{2}]$ | 7 |
| --- | --- | --- |

For $P_{1}=P_{2}$ and defining the spatial period as $\Lambda=\lambda/(2\sin\theta/2)$, Equation 5 can be rewritten including the expression in Equation 7 as follows:

|  | $\left\langle\left. S \right\rangle\right.=\frac{2P}{\pi{w_{o}}^{2}}\exp\left[ -\frac{2}{{w_{o}}^{2}}\left( x^{2}+y^{2}\cos^{2} \frac{\theta}{2}+z^{2}\sin^{2} \frac{\theta}{2} \right) \right]\times\left\{ e_{z}\cos\frac{\theta}{2}\left[ \cosh\left( \frac{2yz\sin\theta}{\sigma^{2}} \right)+\cos\left( \frac{2\pi}{\Lambda} \right) \right]+e_{y}\sin\frac{\theta}{2}\sinh\left( \frac{2yz\sin\theta}{\sigma^{2}} \right) \right\}.$ | 8 |
| --- | --- | --- |

Equation 4 describes the variation of the Poynting flux as a function of the space coordinates $(x,y,z)$ and the interference angle $\theta$. A cross section of the $\left\langle\left. S \right\rangle\right.$ distribution in plane y-z is depicted in Figure S1a, for two interfering beams having $\lambda$ = 1064 nm, $\Lambda$ = 8 µm and $\sigma$= 50 µm, together with the geometrical representation of the beams (dotted lines). As it can be noted, the interference volume resembles an ellipsoid and its borders go beyond the region in which the geometric rays cross over. As a convention, the position in which the geometric rays intersect (Figure S1c) is taken as the zero-reference for the z position, by which the interference assumes its maximum contrast (Figure S1f). At a 0.5 mm over the geometrical interference position ($z$ = 0.5 mm, Figure S1b) the beams are defocused and separate gradually, lowering the interference intensity (Figure S1e). A similar behaviour can be seen at 0.5 mm beyond the interference position ($z$ = -0.5 mm, Figure S1d), where the beams focus and separate while propagating, decreasing the interference contrast (Figure S1g).


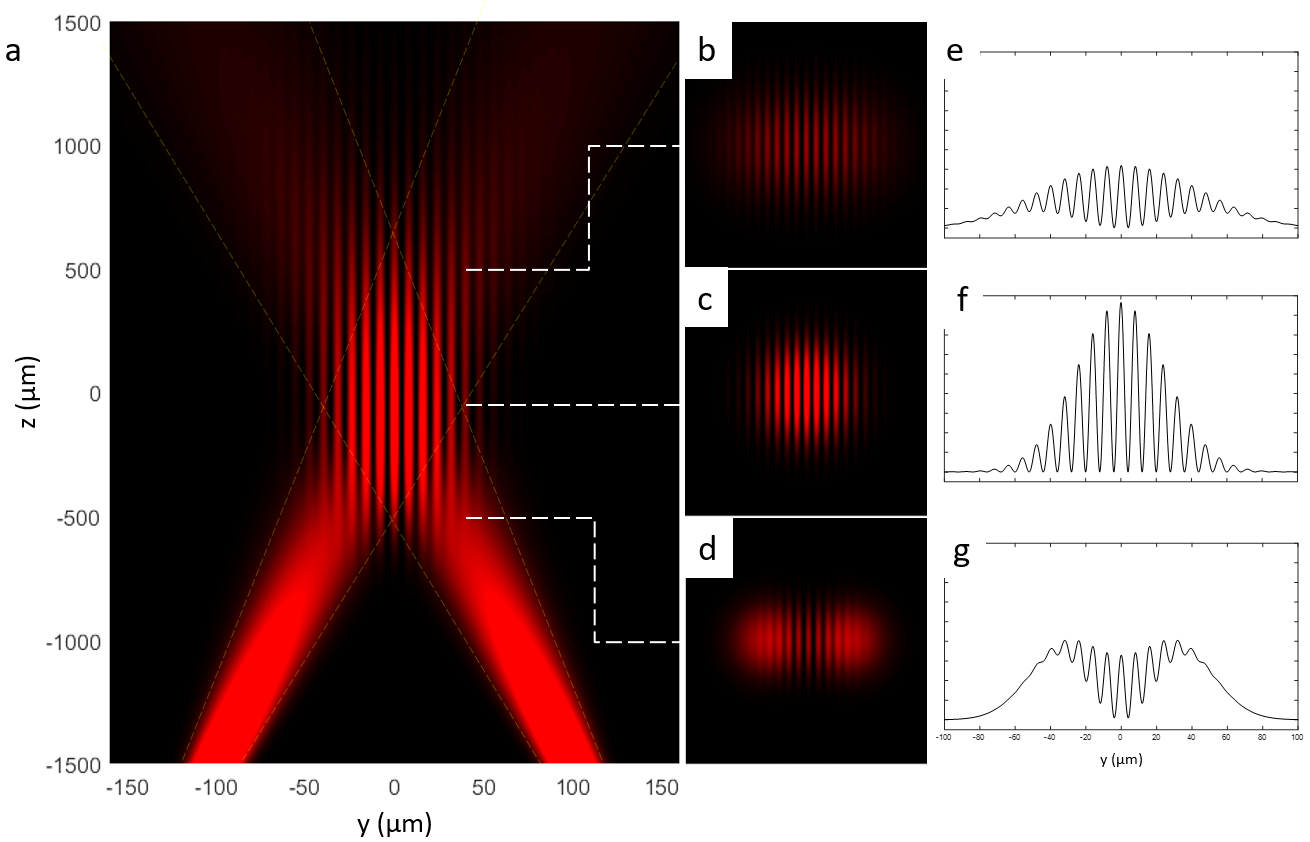


Fig. S1: Cross section of the interference volume in the YZ plane (a), projections in the XY plane at different Z positions (b, c, d) and intensity profile of the respective intensity distributions (e, f, g).

*Spectral analysis*


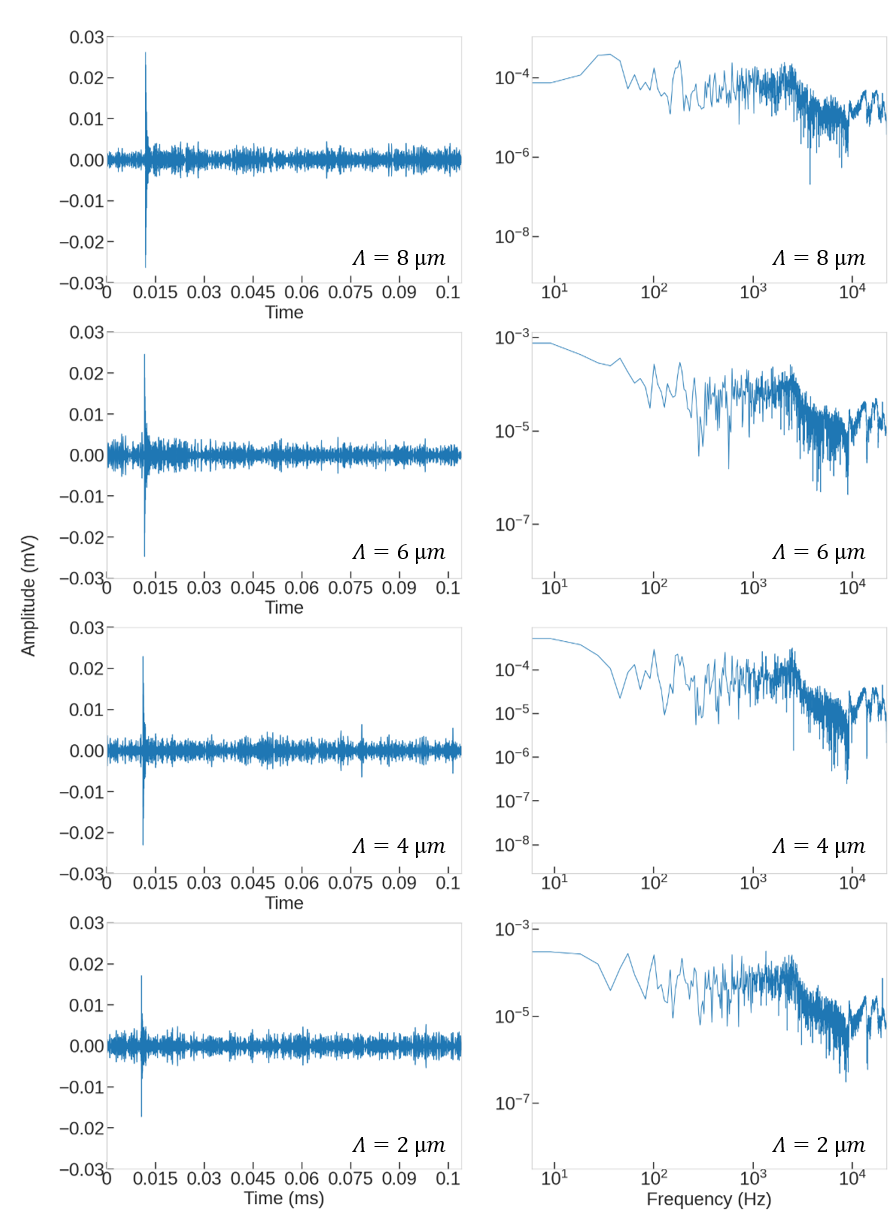


Fig. S2: Change of the AE signals in time for spatial period 8µm, 6µm, 4µm and 2µm and frequency domains

**References**

1. Brayton, D. B. Small Particle Signal Characteristics of a Dual-Scatter Laser Velocimeter. *Applied Optics* **13**, 2346 (1974).
